# Heterometallic ZnHoMOF as a Dual-Responsive Luminescence Sensor for Efficient Detection of Hippuric Acid Biomarker and Nitrofuran Antibiotics

**DOI:** 10.3390/molecules28176274

**Published:** 2023-08-27

**Authors:** Jingrui Yin, Wenqian Li, Wencui Li, Liying Liu, Dongsheng Zhao, Xin Liu, Tuoping Hu, Liming Fan

**Affiliations:** 1 Shanxi Key Laboratory of Advanced Carbon Electrode Materials, Shanxi Coal Mine Water Treatment Technology Innovation Center, School of Chemistry and Chemical Engineering, North University of China, Taiyuan 030051, China; jingruiyinzbdx@163.com (J.Y.);; 2 Key Laboratory of Advanced Energy Materials Chemistry (Ministry of Education), College of Chemistry, Nankai University, Tianjin 300071, China

**Keywords:** antibiotics detection, bifunctional luminescence sensor, biomarker detection, heterometallic metal–organic framework, sensing mechanism

## Abstract

Developing efficient and sensitive MOF-based luminescence sensors for bioactive molecule detection is of great significance and remains a challenge. Benefiting from favorable chemical and thermal stability, as well as excellent luminescence performance, a porous Zn(II)Ho(III) heterometallic–organic framework (ZnHoMOF) was selected here as a bifunctional luminescence sensor for the early diagnosis of a toluene exposure biomarker of hippuric acid (HA) through “turn-on” luminescence enhancing response and the daily monitoring of NFT/NFZ antibiotics through “turn-off” quenching effects in aqueous media with high sensitivity, acceptable selectivity, good anti-interference, exceptional recyclability performance, and low detection limits (LODs) of 0.7 ppm for HA, 0.04 ppm for NFT, and 0.05 ppm for NFZ. Moreover, the developed sensor was employed to quantify HA in diluted urine samples and NFT/NFZ in natural river water with satisfactory results. In addition, the sensing mechanisms of ZnHoMOF as a dual-response chemosensor in efficient detection of HA and NFT/NFZ antibiotics were conducted from the view of photo-induced electron transfer (PET), as well as inner filter effects (IFEs), with the help of time-dependent density functional theory (TD-DFT) and spectral overlap experiments.

## 1. Introduction

The increasing focus on environmental concerns in contemporary society has led to heightened attention toward toluene, an essential volatile solvent in paints and polymer coatings, a crucial organic chemical raw material, and a significant high-octane gasoline additive. Its extensive utilization in the chemical industry and everyday life has been well-documented. Extensive research has demonstrated that toluene not only presents a substantial environmental hazard but also has the potential to diminish human immunity upon prolonged exposure, thereby potentially resulting in childhood leukemia, chronic kidney failure, and irreversible damage to the nervous system [1,2]. In contrast to the variety and practicality of toluene detection methods employed in environmental settings, the options available for detecting absorbed toluene within the human body are limited. Nevertheless, given the inherent disparities among individuals, the detection of toluene levels in humans holds greater significance and relevance than that of toluene in the environment. Research indicates that approximately 80% of absorbed toluene undergoes metabolic transformation into the biomarker known as hippuric acid (HA) in the liver and kidneys, subsequently being excreted through urine [3]. As the exposure biomarker of toluene, the HA level in urine, thus, can well reflect the level of toluene exposure in the human body [4]. Meanwhile, the environmental and health issues caused by antibiotics are going from bad to worse. The judicious utilization of antibiotics is crucial in the management and prevention of diseases, safeguarding the body against bacterial infections and significantly enhancing human well-being. Regrettably, the inappropriate and excessive application of antibiotics in both medical and livestock sectors transforms beneficial practices into detrimental ones. Ultimately, recalcitrant antibiotics accumulate in humans via the water cycle and food chain, resulting in profound adverse effects, substantial impairment of liver and kidney functions, and the emergence of drug-resistant superbugs [5,6]. Considering the cost and broad antibacterial issues, nitrofuran antibiotics, especially nitrofurantoin (NFT) and nitrofurazone (NFZ), are the most commonly used antibiotics in the fields of animal husbandry, poultry farming, and fisheries. Ultimately, the overused or misused nitrofuran antibiotics accumulate in the human body through the water cycle and food chain, leading to serious problems, such as liver and kidney damage, decreased immunity, and increased bacterial resistance [7,8]. Thus, the rapid and sensitive detection of nitrofuran antibiotics in natural water is also crucial. From the viewpoints of human health and environmental protection, it is urgent to design a simple, sensitive, and stable monitoring method for the biomarker, HA, of toluene exposure and nitrofuran antibiotics in water media. Current methods for detecting HA in urine and monitoring nitrofuran antibiotics in water media typically involve colorimetry, gas chromatography, spectrophotometry, high-performance liquid chromatography (HPLC), electrochemical analysis, or a combination of ultra-high-performance liquid chromatography and mass spectrometry (UPLC-MS). These traditional detection methods, while effective, are characterized by the use of cumbersome and expensive instruments, which present operational challenges and result in increased costs and time for detection. Consequently, these factors hinder the feasibility of daily monitoring of antibiotics in water and the early diagnosis of HA in urine [9,10]. To fill this gap, there is an urgent need to develop sensitive, efficient, and environmentally friendly sensors. Benefiting from the advantages of advanced optical technology, an efficient optical detection method, uncomplicated equipment, and visually interpretable outcomes, the rapidly expanding field of fluorescent sensors has emerged as a promising avenue for achieving a significant breakthrough.

As a booming class of organic–inorganic hybrid porous materials, the stable skeleton, larger specific surface area, as well as modifiable active sites, thereby enable their widespread applications in the fields of storage and separation, heterogeneous catalysis, proton conduction, and so on [11,12,13,14,15,16,17,18,19,20]. Leveraging the advantages of multiple emitting sources, MOFs also provide excellent platforms as luminescence sensors in the detection of heavy metal ions, highly oxidized anions, hazardous organic molecules, or even bioactive molecules [21,22,23,24,25,26,27,28]. Returning to the subject, since Zhou and Li reported the first example of an MOF-based sensor for nitrofurazone antibiotic detection through luminescent quenching effects in 2016 [27], more and more MOFs have been designed to achieve more sensitive detection results [29,30]. Turning to MOF-based HA sensors, it is easy to find that there are only seven reports when searching Web of Science [31,32,33,34]. According to the theory of hard and soft acid and base (HSAB), rare-earth ions and transition-metal ions often exhibit distinct coordination preferences in the construction of MOFs. When both rare-earth ions and transition-metal ions were introduced into the framework as inorganic nodes, the self-assembled MOFs not only exhibited novelty in structures but also displayed unique performances. There is no doubt that heterometallic–organic frameworks (HMOFs) have great potential as luminescence sensors in the detection of pollutants. Nevertheless, there are only a few samples of identical HMOF-based chemosensors to detect antibiotics or biomarkers that have been reported up to now [35,36].

**Scheme 1:**
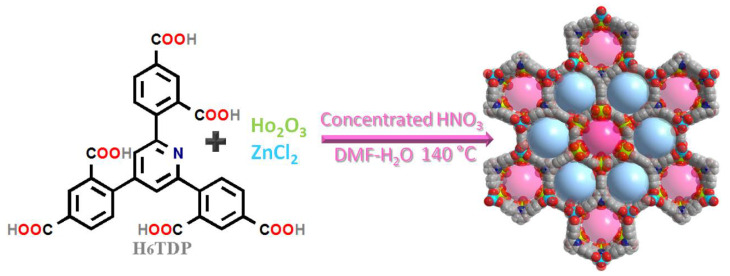
The preparation process of the heterometallic ZnHoMOF.

Our group has been engaged in developing MOF-based sensors for detecting bioactive molecules in recent years [37,38]. Inspired by the above considerations and following our research interests of constructing MOF-based luminescence sensors, herein, an ultra-stable luminescent Zn(II)Ho(III) heterometallic–organic framework of {(Me_2_NH_2_)[ZnHo(TDP)(H_2_O)]·3DMF·3H_2_O}_n_ (ZnHoMOF) was fabricated under solvothermal conditions by using the ligand of 2,4,6-Tri(2′,4′-dicarboxyphenyl)pyridine (H_6_TDP) to react with ZnCl_2_ and Ho_2_O_3_ (Scheme 1). The 3D ZnHoMOF is a {ZnHo(COO)_3_} SBU-based 3D {4^6^·6^4^} **fng** net, in which it contains two kinds of channels and up to 60.4% potential available cavities. Luminescence sensing studies showed that ZnHoMOF is an efficient bifunctional luminescence sensor for HA biomarker detection in diluted urine samples through “turn-on” luminescence enhancing response and NFT/NFZ antibiotics detection in natural water through “turn-off” luminescent quenching effects. Furthermore, sensing mechanisms were also conducted here.

## 2. Result and Discussion

### 2.1. Simple Structural Description of ZnHoMOF

**Figure 1:**
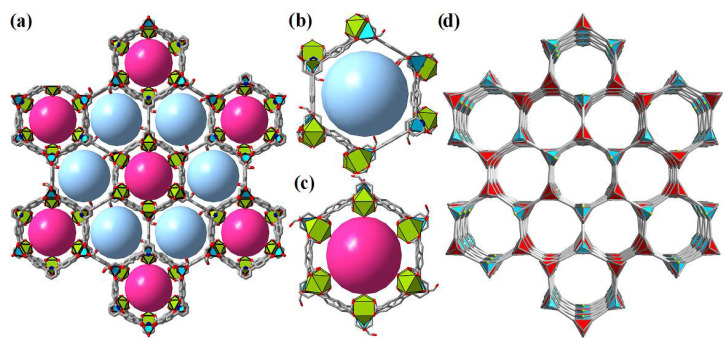
(**a**) The 3D anionic framework of ZnHoMOF. (**b**) Channel A decorated by the uncoordinated carboxyl O atoms. (**c**) Channel B with the coordination-unsaturated metal cations. (**d**) Heterometallic binuclear {ZnHo(COO)_3_} SBU-based 3D {4^6^·6^4^} **fng** topology of ZnHoMOF.

To better understand the structure of ZnHoMOF, its crystal structure was analyzed in detail here. ZnHoMOF is an anionic porous framework (Figure 1a) that contains two kinds of channels. The aperture of channel A is about 12.8 Å, with the inter surface decorated by uncoordinated carboxyl O atoms (Figure 1b), while channel B holds an aperture of about 11.4 Å, with coordination-unsaturated metal cations evenly located on the inner surface (Figure 1c). The total potential available volume of ZnHoMOF is 15,979.4 Å^3^, 60.4% of per-unit cell volume (26,443.9 Å^3^). The host framework of ZnHoMOF can be simplified into a uninodal five-connected **fng** net with a point (Schlafli) symbol of {4^4^.6^2^} (Figure 1d). The large porosity as well as the size-suitable channels of ZnHoMOF provide a place for host–guest interactions during the sensing process, while the complicated inter surfaces of those channels provide a variety of active sites, which can further deepen the host–guest interaction intensity.

### 2.2. Chemical and Thermal Stability of ZnHoMOF

As we all know, material practicality depends on chemical stability. Therefore, the chemical stability of ZnHoMOF was checked firstly by immersion in water under different acid and alkali intensities, with pH ranging from 3 to 11 for 6 h. A PXRD profile comparison illustrated ZnHoMOF possessing excellent chemistry stability under the aforementioned harsh conditions (Figure S1). Moreover, the thermal stability of sensing materials also determines, to some extent, the scenarios in which they are used. The thermal stabilities of the as-synthesized and the activated ZnHoMOF samples were investigated in the range of room temperature to 980 °C by using TG-DTG simultaneous measurements under a N_2_ atmosphere (Figure S2). The results show that the first weight loss at 50–230 °C corresponds to the departure of the guest and coordinated molecules; then, the following platform illustrates that the main framework of ZnHoMOF can be well maintained until the temperature reaches 390 °C.

### 2.3. Detection of the HA

**Scheme 2:**
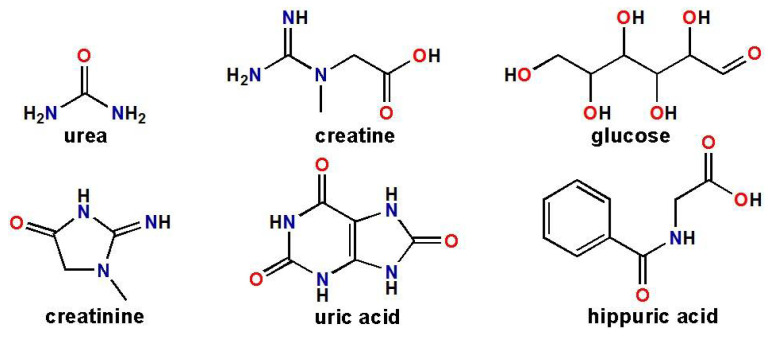
Chemical structures of selected urine chemicals as analytes in this work.

**Figure 2:**
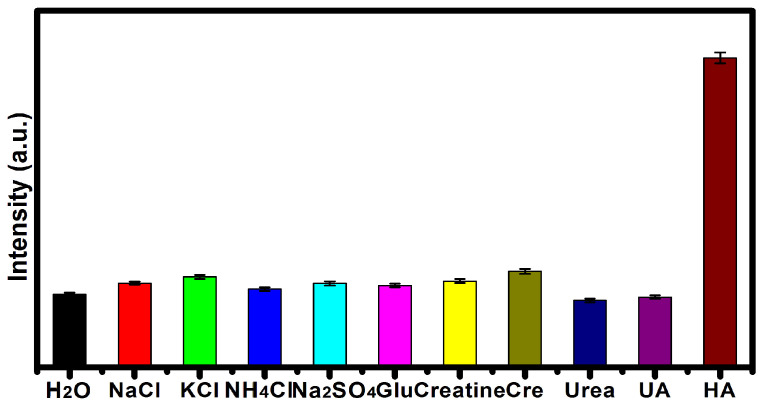
Luminescence intensities of ZnHoMOF dispersed in 0.01 M urine chemical aqueous solutions upon excitation at 325 nm.

The biomarkers, which reflect the changes in organs, tissues, and cells, have a very wide range of applications in the fields of disease diagnosis, disease staging, or evaluating the safety and effectiveness of new drugs [39,40]. As the exposure biomarker of toluene, HA is usually metabolized from the intake of toluene by the kidney and liver and then excreted in urine. Thus, it is suitable and convenient to monitor the HA level in urine to reflect the absorbed toluene in the human body. Before the luminescent sensing of the HA biomarker, the solid-state luminescence properties of ZnHoMOF, as well as the free H_6_TDP ligand, were first tested with an excitation wavelength of 325 nm at room temperature. As shown in Figure S3, the samples of ZnHoMOF exhibit strong blue emission at 406 nm, blue-shifted 60 nm from the emission peak of free H_6_TDP, which is predominantly due to the metal-to-ligand charge transfer (MLCT) [41,42,43]. After being fully grounded and evenly dispersed in the aqueous solutions of urine chemicals, such as (sodium chloride (NaCl), sodium sulfate (Na_2_SO_4_), potassium chloride (KCl), ammonium chloride (NH_4_Cl), urea, glucose (Glu), uric acid (UA), creatinine (Cre), creatine, and HA, Scheme 2) using ultrasonic treatment, the ZnHoMOF suspensions were transferred to detect the luminescent spectra. The results show that the HA displayed more than a quadruple fluorescence enhancement, while the other urine chemicals exhibited little to no changes in the luminescent intensities (Figure 2 and Figure S4).

**Figure 3:**
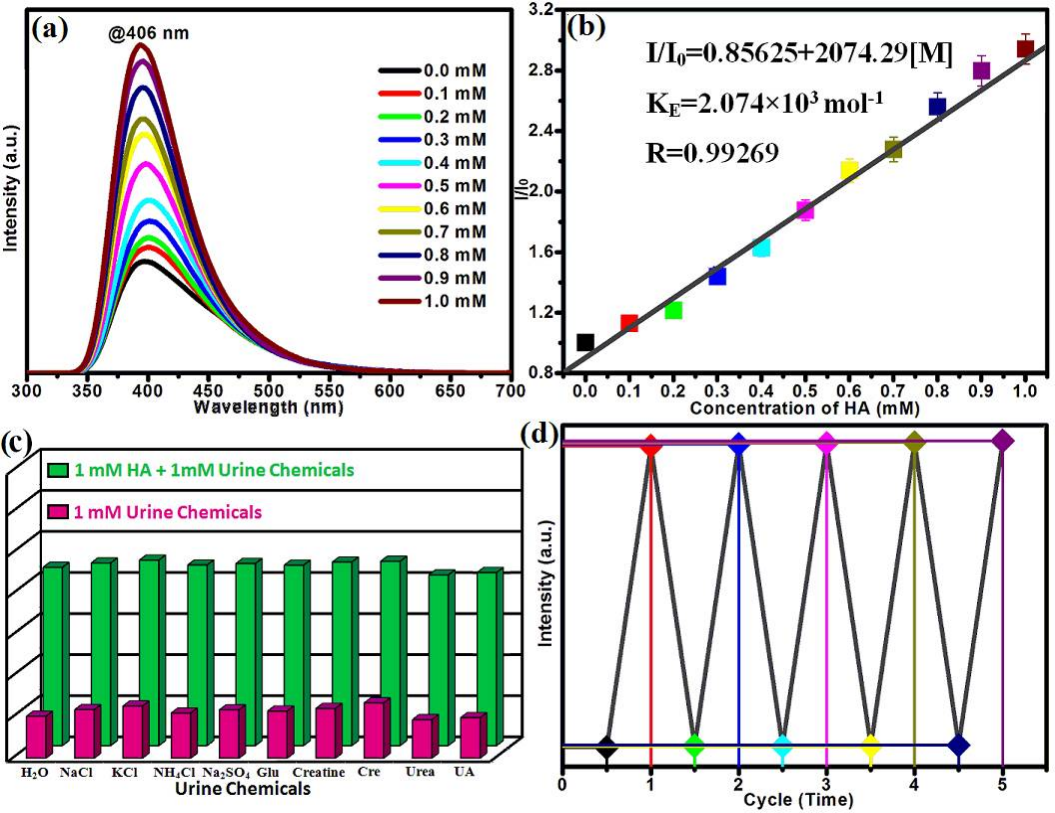
(**a**) The enhanced emission spectra of ZnHoMOF in water with the incremental addition of HA biomarker. (**b**) The relationship between the luminescent intensities of ZnHoMOF suspensions and the concentrations of HA biomarker. (**c**) The anti-interference performance and (**d**) recyclable behavior of ZnHoMOF when sensing HA biomarker.

To further learn the intrinsic relationships between the luminescent intensities and the concentrations of HA, series titration trials were carried out by gradually adding 10 mM HA to the testing aqueous solutions. A comparison of the fluorescence spectra showed that the intensities of ZnHoMOF suspensions were remarkably enhanced with the concentrations of HA increasing (Figure 3a and Figure S5). Based on the results of gradient experiments, the relationship between the concentration of HA and the intensities of ZnHoMOF suspensions was obtained and could be well fitted with a linear equation of *I*/*I*_0_ = 2074.29[M] + 0.85625 (*I*/*I*_0_ = 1 + *K*_E_[M]) (Figure 3b), with the obtained *K*_E_ equivalent or even better than the reported ones (Table 1) [33,34]. On the basis of the equation of LOD = 3*σ*/*K*_E_ (here, *σ* is the standard deviation for ten cycles of blank luminescence tests), the limit of detection (LOD) of ZnHoMOF for detecting HA is about 0.7 ppm. Moreover, it should be pointed out that the fitting equation exhibits large errors at lower concentrations (0–0.05 mM), and the actual linear detection range is 0.05–1.0 mM. According to ACGIH, the recommended standard of the biological exposure limit of HA in urine is 2.0 mg mL^−1^, indicating ZnHoMOF holds great potential in the detection of HA in actual urine.

ZnHoMOF is an excellent fluorescence sensor with high sensitivity for the detection of the HA biomarker. Meanwhile, the anti-interference performances of other urine chemicals were also investigated to research the selectivity of ZnHoMOF to detect the HA biomarker. When 10 mM disturbed urine chemicals were injected into the dispersions of ZnHoMOF containing 1 mM HA, the luminescence intensities of these dispersions only showed slight changes. By comparing the red and the green cylinders in Figure 3c, the results showed that the disturbed urine chemicals only have minimal effects on the detection of HA, confirming that ZnHoMOF shows high selectivity in detecting HA, even in the presence of disturbed urine chemicals. Moreover, recyclability, as a significantly important factor in practical application, was also explored here by using the recycled crystalline samples of ZnHoMOF to retest the luminescent sensing for the HA biomarker for five cycles (Figure 3d). The results indicated that the luminescent property of ZnHoMOF can be well maintained, and ZnHoMOF exhibits extraordinary stability and remarkable recyclability.

We further explored the possibility of detecting HA in real human urine samples, a more complex physiological environment. Urine samples were collected from healthy volunteers; after being centrifuged and diluted 1000-fold, the samples were prepared by adding different amounts of HA. Considering that the actual linear detection range is 0.05–1.0 mM, here, three parallel experiments of 50, 100, 150, and 200 μM were performed to conduct a comprehensive evaluation of relative standard deviations (RSDs) and recovery. As expected, the luminescence intensities of treated suspensions were significantly quenched with an increase in HA concentrations. The results of the internal standard method are given in Table 2. Considering that the actual urine contains a certain amount of HA [44,45], to accurately reflect the actual detection effect, the actual concentration of HA in 1000-fold-diluted urine was measured using the HPLC method, and the mean value of 2.38 μM (n = 3) was used to calculate the following recoveries. High recoveries of 96.7–101.0% as well as the low relative standard deviations (RSDs) in a range of 1.53–2.56% both indicated that ZnHoMOF can serve as a chemosensor for detection in real samples.

### 2.4. Detection of Nitrofuran Antibiotics

**Scheme 3:**
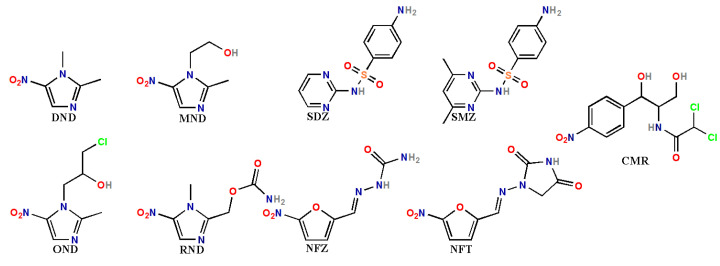
Chemical structures of selected antibiotics as analytes in this work.

**Figure 4:**
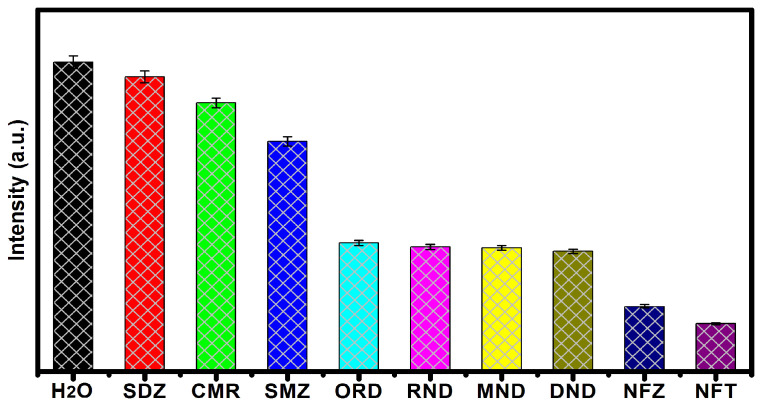
Luminescence intensities of ZnHoMOF dispersed in 0.1 mM antibiotic aqueous solutions upon excitation at 325 nm.

Antibiotics are an important class of drug molecules, serving as security guards to protect humans from harmful viruses, bacteria, and fungi. However, the overuse and misuse of antibiotics often lead to high doses of antibiotics being enriched in our bodies and seriously endangering our human health. The nine most commonly used antibiotics, including sulfamethazine (SMZ), chloramphenicol (CMR), sulfadiazine (SDZ), ronidazole (RND), 1,2-dimethyl-5-nitroimidazole (DND), metronidazole (MND), nitrofurantoin (NFT), ornidazole (OND), and nitrofurazone (NFZ), were selected here as the representatives of antibiotics (Scheme 3). Different from the HA biomarker, the antibiotics show different degrees of “turn-off” effects in the fluorescence of ZnHoMOF suspensions. The order of quenching rates is NFT > NFZ > DND > MND > RND > OND > SMZ > CMR > SDZ, indicating that nitrofuran antibiotics show stronger fluorescence quenching effects than nitroimidazole antibiotics and other antibiotics. The above-mentioned results demonstrated that ZnHoMOF can sense nitrofuran antibiotics with remarkable selectivity (Figure 4 and Figure S6).

**Figure 5:**
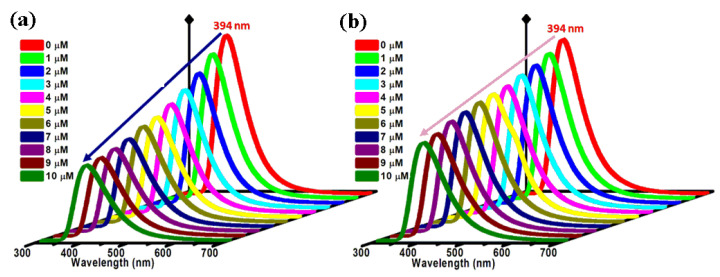
Emission spectra of ZnHoMOF in aqueous solutions containing different amounts of NFT (**a**) and NFZ (**b**).

**Figure 6:**
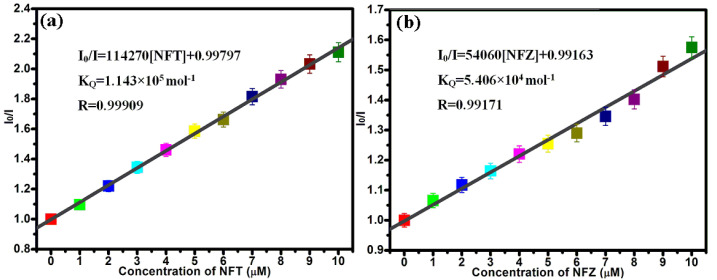
The *I*_0_*/I* of ZnHoMOF suspensions versus the concentrations of NFT (**a**) and NFZ (**b**).

Then, the quantitative detection of nitrofuran antibiotics was also conducted through a series of gradient tests. As shown in Figure 5 and Figure S7, serial titration experiments proved that the luminescence intensities of ZnHoMOF steadily decreased when nitrofuran antibiotics were continuously added. There are linear correlations between the intensities of ZnHoMOF and the concentrations of nitrofuran antibiotics, which both fit well with the Stern–Volmer equations of *I*_0_/*I* = 114,270[NFT] + 0.99797 for NFT and *I*_0_/*I* = 54,060[NFZ] + 0.99163 for NFZ (Figure 6). The R values of two linear fitting equations indicated that the detection ranges of ZnHoMOF in detecting NFT/NFZ antibiotics are both 0–10 μM. Calculated by LOD = 3*σ*/*S*, where S means K_Q_ here, the K_Q_ and the LOD values are 1.143 × 10^5^ mol^−1^ and 0.04 ppm for NFT and 5.406 × 10^4^ mol^−1^ and 0.05 ppm for NFZ, respectively, which are comparable to or better than those of some reported MOFs [46,47,48,49] (Table 3). Moreover, the recyclable experiments of ZnHoMOF for the sensing of nitrofuran antibiotics were also performed. The results demonstrated that the quenching effects of two nitrofuran antibiotics on the luminescent intensities of ZnHoMOF showed no significant decrease after five cycles (Figure S8). It should be pointed out that other antibiotics, especially nitro-containing antibiotics, also have a certain degree of luminescence quenching with respect to the luminescence intensity of the ZnHoMOF, which will inevitably lead to a decrease in selectivity in the actual detection process. Moreover, when NFT and NFZ antibiotics exist simultaneously in the aqueous solutions, they will affect each other, leading to a significant deviation in the detection results. Considering that the actual samples, especially the diluted samples, may contain very few antibiotics, the selectivity is, thus, acceptable here. These performances demonstrated that ZnHoMOF is an efficient luminescence sensor in detecting aqueous nitrofuran antibiotics in laboratory environments.

In order to achieve practical application in real scenarios, the detection capability of NFT/NFZ in Fen River water was investigated. The fluorescence titration experiments were carried out by adding the powdered ZnHoMOF samples into the filtered Fen River water and were well dispersed to prepare the suspensions. Here, the NFT/NFZ concentrations in the Fenhe River water determined using the HPLC method were close to 0. Thus, the added concentrations of above-mentioned antibiotics were considered as the actual concentrations. By adopting an internal standard method and conducting three tests, a series of results were achieved with satisfactory recoveries (99.33–103.00% for NFT and 98.50–105.00% for NFZ) as well as low RSDs (1.88–2.16% for NFT, 1.87–2.17% for NFZ), as shown in Table 4; both indicated that ZnHoMOF can serve as a luminescence sensor for detecting NFT/NFZ antibiotics in real natural waters.

### 2.5. Possible Mechanism for Luminescence Sensing

**Figure 7:**
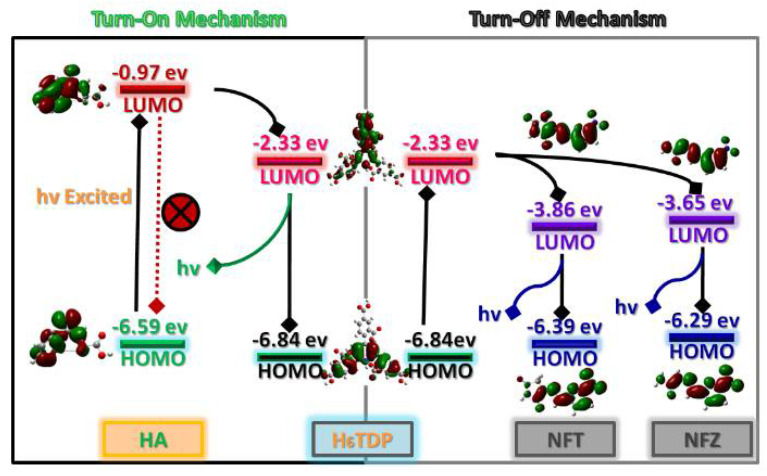
Mechanisms of “turn−on” sensing for HA and “turn−off” sensing for NFT/NFZ from the viewpoint of photo-induced electron transfer (PET).

**Figure 8:**
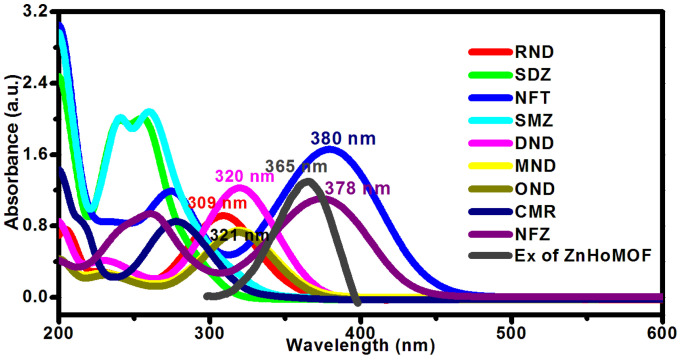
UV-Vis absorbance spectra of antibiotics in aqueous solutions, as well as the excitation spectrum of ZnHoMOF.

A deeper understanding of the sensing mechanisms is crucial for further research and the development of more effective luminescence sensors. Prior studies have indicated that luminescent quenching effects are mainly caused by electron and energy transfer, as well as structure collapse [50,51]. As the first and foremost evaluation indicator, the stability of frameworks plays a crucial role and directly determines the actual values and application scenarios [52,53,54,55]. Fortunately, the structure collapse of ZnHoMOF can be excluded by comparing the PXRD profiles of reused crystalline materials with the original ones (Figure S9). Brunauer–Emmett–Teller (BET) tests of the recycled ZnHoMOF samples also proved that the pore structure of ZnHoMOF was well maintained during the sensing process (Figure S10). Moreover, comparisons of the ZnHoMOF IR spectra before and after the sensing process also confirmed the stability of the framework (Figure S11). Secondly, the luminescence decay lifetimes of the ZnHoMOF samples before and after sensing nitrofuran antibiotics (NFT and NFZ) were further measured and performed, as shown in Figure S12, with only a slight change compared with the original one, indicating the occurrence of the static quenching process. Subsequently, the mechanisms underlying the luminescence response of the photo-induced electron transfer (PET) in ZnHoMOF were investigated through theoretical calculations. It was observed that the excited electrons were transferred from the lowest unoccupied molecular orbitals (LUMO) of ZnHoMOF to the LUMO of nitrofuran antibiotics, rather than its highest occupied molecular orbitals (HOMO). This transfer resulted in the “turn-off” effect of ZnHoMOF’s luminescence. Additionally, the excited electrons were found to transfer from the LUMO of HA to the LUMO of ZnHoMOF, leading to the “turn-on” effect of ZnHoMOF’s luminescence [56,57]. This conjecture was verified by calculating the LUMO and HOMO energy levels of HA, NFT/NFZ antibiotics, and free H_6_TDP with the help of Gaussian 09 software at a 3LYP/6-31 + G* theoretical level. Figure 7 illustrates that the LUMO energy levels of nitrofuran antibiotics are lower than that of the H_6_TDP ligand, and the HOMO energy level of HA is higher than that of the H_6_TDP ligand, giving the answer to the “turn-on” enhancing effect of HA and the “turn-off” quenching effects of nitrofuran antibiotics on the luminescence of ZnHoMOF. Finally, inner filter effects (IFEs) also play a crucial role in the nitrofuran-antibiotics-caused quenching processes. As displayed in Figure 8, a large overlap exists between the absorption spectra of the NFT/NFZ antibiotics and the excitation spectrum of ZnHoMOF, implying that the UV-visible absorption of nitrofuran antibiotics hinders the normal excitation energy transfer process of ZnHoMOF, which causes light-competitive absorption [58,59].

## 3. Materials and Methods

### 3.1. Reagents and Apparatus

The 2,4,6-Tri(2′,4′-dicarboxyphenyl)pyridine (H_6_TDP) ligand and holmium oxide (Ho_2_O_3_) were bought from Jinan Henghua Technology Co., Ltd. (Jinan, China); the organic small biological molecules (including urea, creatine, Glu, Cre, and HA) and antibiotics (including SDZ, MND, SMZ, CMR, OND, RND, DND, NFZ, and NFT) were purchased from Shanghai Macklin Biochemical Technology Co., Ltd. (Shanghai, China); and the metal salts (NaCl, Na_2_SO_4_, KCl, NH_4_Cl, and ZnCl_2_), N,N-dimethylformamide (DMF), and concentrated HNO_3_ were purchased from Aladdin Chemistry Co., Ltd. (Shanghai, China). All reagents were commercially obtained for direct use with no further purification. A Nicolet NEXUS-670 FT-IR spectrometer (Thermo Nicolet Corporation, Madison, WI, United States) was used to record the FT-IR spectrum by using the KBr pellet. The PXRD patterns were obtained using a RigakuD/Max-2500 PC diffractometer (Rigaku Corporation, Tokyo, Japan) with Cu-Kα radiation (λ = 1.54056 Å). A Hitachi U-5100 spectrophotometer was used to measure UV/Vis absorption spectra. A PerkinElmer DTA 6000 thermogravimetric analyzer (Platinum Elmer Inc., Waltham, MA, USA) was used to carry out thermogravimetric analyses (TGA) at 980 °C at 10 °C per minute under nitrogen conditions. The luminescence spectra and luminescent lifetime were recorded by using a Hitachi F-4600 spectrophotometer and Edinburgh FS5 spectrophotometer, respectively. HPLC results were obtained from a Shimadzu Prominence LC-20A liquid chromatograph (Shimadzu Scientific Instruments, Inc., Kyoto, Japan). BET surface areas were measured at 77 K via N_2_ adsorption/desorption by using an ASAP 2020 Plus instrument (Micromeritics Instrument. Corp., Atlanta, GA, USA).

### 3.2. Preparation of ZnHoMOF

ZnHoMOF was prepared according to a previously reported study [60]. In the feasible synthesis of ZnHoMOF, 0.10 mmol ZnCl_2_·H_2_O, 0.05 mmol Ho_2_O_3_, 0.06 mmol H_6_TDP, 0.2 mL concentrated HNO_3_, 2 mL H_2_O, and 7 mL DMF were mixed and thoroughly stirred in a 25 mL autoclave. Colorless crystals of ZnHoMOF were prepared by heating at 140 °C for 96 h, then gradually cooling to room temperature at 10 °C per hour. The fresh ZnHoMOF samples were obtained after further filtration, cleaning, and drying. FT-IR (cm*^−^*^1^): 3441 (vs), 2356 (s), 1655 (vs), 1601 (s), 1559 (s), 1389 (vs), 1251 (m), 1102 (w), 831 (w), 788 (m), 672 (w), 480 (w).

### 3.3. Luminescent Sensing Experiments

A thorough grinding of the prepared samples was required before the sensing tests could be conducted. Then, 2 mg well-ground samples of ZnHoMOF were added to 2 mL aqueous solutions containing 0.01 M urine chemicals (NaCl, Na_2_SO_4_, KCl, NH_4_Cl, urea, creatine, Glu, Cre, and HA) or 0.1 mM antibiotics (SDZ, MND, SMZ, CMR, OND, RND, DND, NFZ, and NFT). After ultrasonic treatment for 30 min, the prepared suspensions were transferred to a 1 cm width quartz cell to achieve fluorescence emission spectra.

### 3.4. Luminescent Titration and Recyclable Experiments

To further explore internal relationships between the concentrations of analytes and fluorescence intensities, luminescent titrations were conducted by adding different amounts of analytes to 2 mL suspensions of ZnHoMOF (2 mg) in quartz cell; then, the emission spectra of suspensions were tested. Next, the ZnHoMOF sample was processed through filtration, cleaning, and drying processes to obtain the recovered sample and underwent subsequent gradient testing in the next cycle. Moreover, the anti-interference performances of analytes were also checked by testing the emission spectra of the dispersions of ZnHoMOF in a certain amount of analytes and the mixture dispersions of equimolar analytes and analytical interferences in sequence.

## 4. Conclusions

In summary, a chemo-robust luminescent Zn(II)Ho(III) heterometallic –organic framework was chosen as the luminescence sensor here based on its robust framework in harsh environments and excellent luminescence. Benefiting from the photo-induced electron transfer (PET) from HA to the framework, the prepared ZnHoMOF exhibited an unprecedented “turn-on” response to HA in urine with high sensitivity and selectivity and a low LOD of 0.7 ppm. Meanwhile, it can also act as an efficient sensor in sensitively detecting NFT/NFZ antibiotics with acceptable selectivity, good anti-interference, exceptional recyclability performance, large detection ranges (0–10 μM), and low LODs of 0.04 ppm for NFT and 0.05 ppm for NFZ. Furthermore, the luminescence quenching mechanism of NFT/NFZ toward ZnHoMOF can be attributed to the synergistic effects of photo-induced electron transfer (PET) and inner filter effects (IFEs). The high recoveries of the developed ZnHoMOF sensor in quantifying the HA biomarker and NFT/NFZ antibiotics in real samples provided a meaningful sample in developing efficient and sensitive MOF-based luminescence sensors for bioactive molecule detection.

## Figures and Tables

**Table 1:** Comparison of MOF-based “turn-on” sensors for HA detection in aqueous media.

MOFs	Detection Signal	*K*_E_ (M^−1^)	LODs	Reference
[Co_3_(ndc)(HCOO)_3_(*μ*_3_-OH)]	421 nm	1.219 × 10^3^	4.6 ppm	[33]
[Ni(H_2_TPTA)(bimb)]	441 nm	8.22 × 10^3^	4.1 ppm	[34]
ZnHoMOF	406 nm	2.074 × 10^3^	0.7 ppm	This work

**Table 2:** ZnHoMOF results for quantitatively detecting HA in 1000-fold-diluted urine samples (n = 3).

Added (μM)	Found (μM)	RSD (%)	Recovery (%)
50	50.63	2.23	96.7
100	102.19	1.68	99.8
150	153.87	1.53	101.0
200	198.73	2.56	98.2

**Table 3:** Comparison of MOF-based “turn−off” sensors for NFT/NFZ detection in aqueous media.

MOFs	Analyte	*K*_Q_ (M^−1^)	LODs	Reference
[Cd(tptc)_0.5_(bpz)]	NFT	3.87 × 10^4^	0.06 ppm	[46]
[Cd(tptc)_0.5_(bpy)]	NFT	7.63 × 10^3^	0.18 ppm	[46]
[Cd_10_(DDB)_4_(bpz)_8_]	NFT	1.88 × 10^5^	0.02 ppm	[47]
[Zn(tptc)_0.5_(bimb)]	NFT	1.273 × 10^5^	0.02 ppm	[48]
[Zn(NH_2_−TCB)]	NFT	6.59 × 10^4^	0.07 ppm	[49]
ZnHoMOF	NFT	1.143 × 10^5^	0.04 ppm	This work
[Cd_10_(DDB)_4_(bpz)_8_]	NFZ	1.05 × 10^5^	0.03 ppm	[47]
[Zn (tptc)_0.5_(bimb)]	NFZ	9.556 × 10^4^	0.03 ppm	[48]
[Zn(NH_2_−TCB)]	NFZ	4.85 × 10^4^	0.06 ppm	[49]
ZnHoMOF	NFZ	5.406 × 10^4^	0.05 ppm	This work

**Table 4:** ZnHoMOF results for quantitative detecting NFT/NFZ in real Fen River water (n = 3).

Analyte	Added (μM)	Found (μM)	RSD (%)	Recovery (%)
NFT	1	1.03	2.04	103.0
	2	1.99	1.88	99.5
	3	2.98	2.15	99.9
	4	3.97	2.16	99.3
	5	5.02	2.12	100.4
NFZ	1	1.05	2.05	105.0
	2	1.97	1.87	98.5
	3	2.96	2.17	98.7
	4	3.95	2.13	98.8
	5	5.04	2.08	100.8

## Data Availability

Data will be made available upon reasonable request.

## Supplementary Materials

The following supporting information can be downloaded at: https://www.mdpi.com/article/10.3390/molecules28176274/s1, Figure S1: PXRD patterns of ZnHoMOF after soaking in different pH values; Figure S2: TG DTG curves of as synthesized and activated ZnHoMOF samples; Figure S3: Luminescent spectra of free H_6_TDP and ZnHoMOF in solid state at room temperature; Figure S4: Luminescence of ZnHoMOF dispersed in 0.01 M urine chemicals aqueous solutions; Figure S5: Enhanced emission spectra of ZnHoMOF in water with the incremental addition of HA biomarker; Figure S6: Luminescence of ZnHoMOF dispersed in the 0.1 mM antibiotics aqueous solutions; Figure S7: Emission spectra of ZnHoMOF in aqueous solutions with incremental addition of NFZ (a), and NFT (b); Figgure S8: Recyclable behavior of ZnHoMOF when sensing of NFZ (a), and NFT (b); Figure S9: PXRD patterns of recycled ZnHoMOF after sensing HA, NFT, and NFZ; Figure S10: The luminescence decay lifetimes of ZnHoMOF samples before and after sensing nitrofuran antibiotics; Figure S11: The FTIR spectra of ZnHoMOF before or after sensing of HA, NFT and NFZ; Figure S12: The BET tests of ZnHoMOF before or after sensing of HA, and nitrofuran antibiotics (NFT and NFZ) at 77 K.

## Sample Availability

Samples of the compounds are available from the authors upon reasonable request.
