# Supplementary material for: Heterometallic ZnHoMOF as a Dual-Responsive Luminescence Sensor for Efficient Detection of Hippuric Acid Biomarker and Nitrofuran Antibiotics
Source: Molecules. 2023 Aug 27;28(17):6274. doi: 10.3390/molecules28176274 (PMC10488516; doi:10.3390/molecules28176274)
Supplement: Supplementary file 1 [file molecules-28-06274-s001.zip › molecules-2539124-supplementary.pdf]

Electronic Supplementary Information (ESI) for

Heterometallic ZnHoMOF as A Dual-responsive Luminescence sensor for Efficient Detection of Hippuric Acid Biomarker and Nitrofuran Antibiotics

Jingrui Yin[1], Wenqian Li[1], Wencui Li[1], Liying Liu[1], Dongsheng Zhao[1], Xin Liu[1], Tuoping Hu[1*], and Liming Fan[1,2*]

[1] Shanxi Key Laboratory of Advanced Carbon Electrode Materials, Shanxi Coal Mine Water Treatment Technology Innovation Center, School of Chemistry and Chemical Engineering, North University of China, Taiyuan 030051, P. R. China.

[2] Key Laboratory of Advanced Energy Materials Chemistry (Ministry of Education), College of Chemistry, Nankai University, Tianjin 300071, P. R. China.

E-mail: hutuopingsx@126.com (T. Hu); limingfan@nuc.edu.cn (L. Fan).

Table of Contents

Figure S1. PXRD patterns of ZnHoMOF after soaking in different pH values........2

Figure S2. TG-DTG curves of as-synthesized and activated ZnHoMOF samples........2

Figure S3. Luminescent spectra of free H_6TDP and ZnHoMOF in solid state at room temperature........2

Figure S4. Luminescence of ZnHoMOF dispersed in 0.01 M urine chemicals aqueous solutions........3

Figure S5. Enhanced emission spectra of ZnHoMOF in water with the incremental addition of HA biomarker........3

Figure S6. Luminescence of ZnHoMOF dispersed in the 0.1 mM antibiotics aqueous solutions........3

Figure S7. Emission spectra of ZnHoMOF in aqueous solutions with incremental addition of NFZ (a), and NFT (b)..4

Figure S8. Recyclable behavior of ZnHoMOF when sensing of NFZ (a), and NFT (b)........4

Figure S9. PXRD patterns of recycled ZnHoMOF after sensing HA, NFT, and NFZ........4

Figure S10. The luminescence decay lifetimes of ZnHoMOF samples before and after sensing nitrofuran antibiotics (NFT and NFZ)........4

Figure S11. The FT-IR spectra of ZnHoMOF before or after sensing of HA, NFT and NFZ........5

Figure S12. The BET tests of ZnHoMOF before or after sensing of HA, and nitrofuran antibiotics (NFT and NFZ) at 77K........5

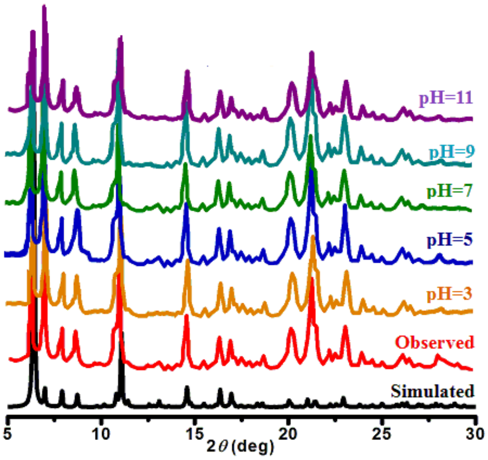

Figure S1. PXRD patterns of ZnHoMOF after soaking in different pH values.

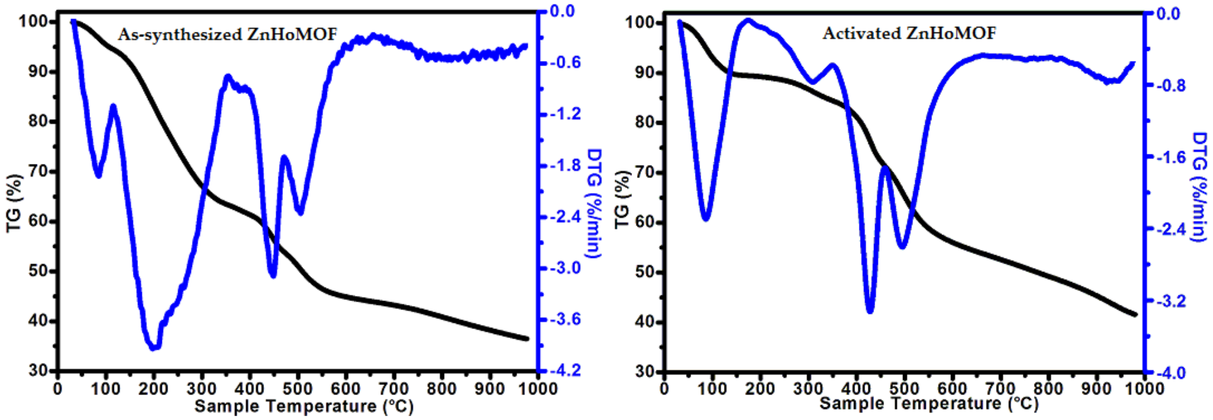

Figure S2. TG-DTG curves of as-synthesized and activated ZnHoMOF samples.

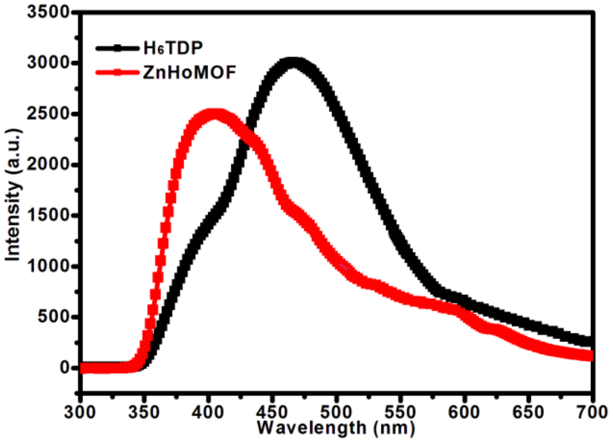

Figure S3. Luminescent spectra of free H_6TDP and ZnHoMOF in solid state at room temperature.

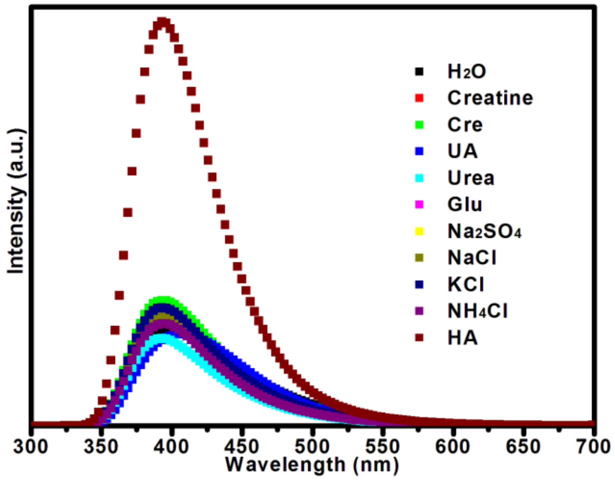

Figure S4. Luminescence of ZnHoMOF dispersed in 0.01 M urine chemicals aqueous solutions.

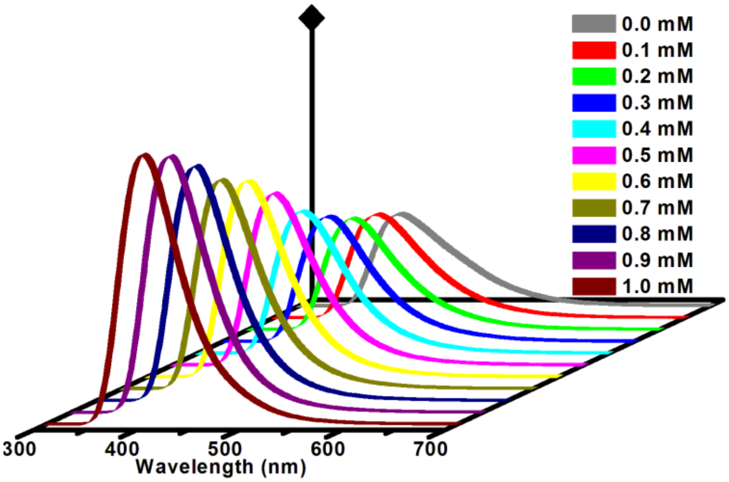

Figure S5. Enhanced emission spectra of ZnHoMOF in water with the incremental addition of HA biomarker.

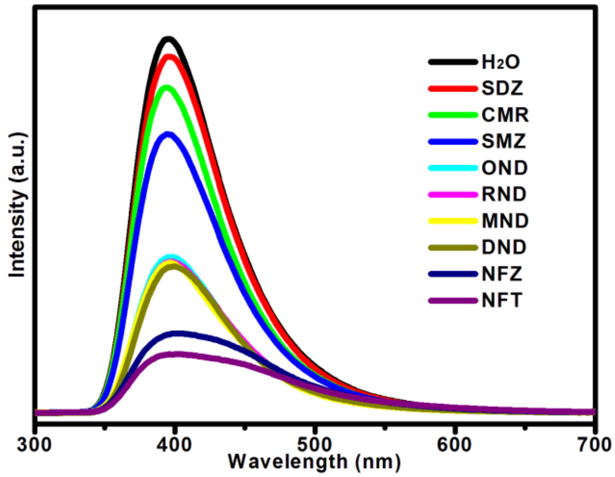

Figure S6. Luminescence of ZnHoMOF dispersed in the 0.1 mM antibiotics aqueous solutions.

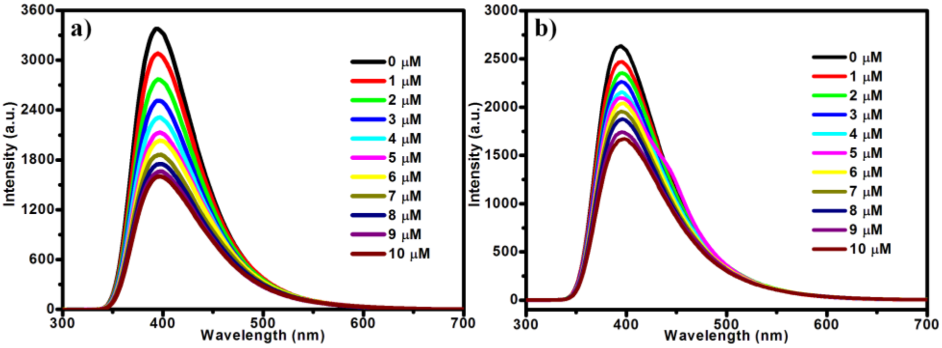

Figure S7. Emission spectra of ZnHoMOF in aqueous solutions with incremental addition of NFZ (a), and NFT (b).

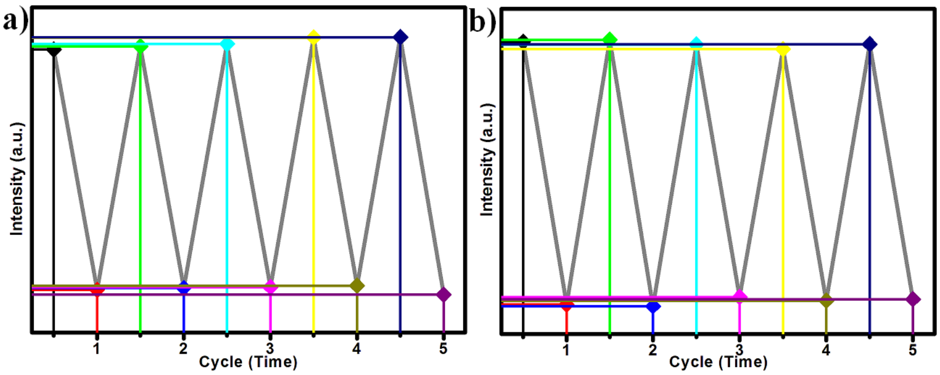

Figure S8. Recyclable behavior of ZnHoMOF when sensing of NFZ (a), and NFT (b).

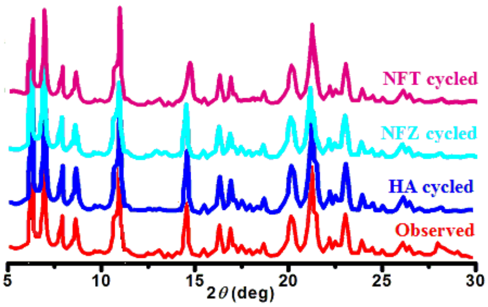

Figure S9. PXRD patterns of recycled ZnHoMOF after sensing HA, NFT, and NFZ.

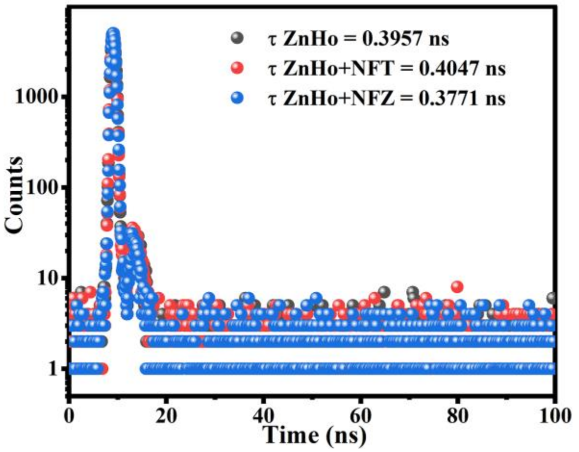

Figure S10. The luminescence decay lifetimes of ZnHoMOF samples before and after sensing nitrofuran antibiotics (NFT and NFZ).

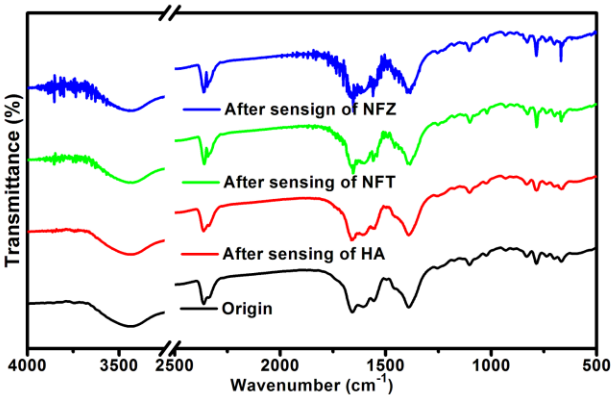

Figure S11. The FT-IR spectra of ZnHoMOF before or after sensing of HA, NFT and NFZ.

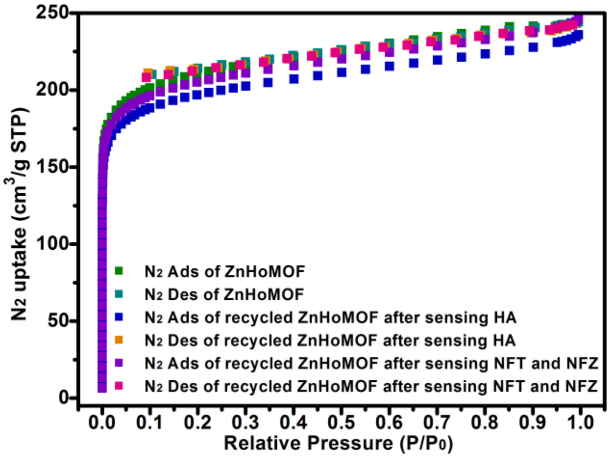

Figure S12. The BET tests of ZnHoMOF before or after sensing of HA, and nitrofuran antibiotics (NFT and NFZ) at 77K.
